# Comparative Outcomes of Suprapectoral and Subpectoral Biceps Tenodesis: A Systematic Review of Fixation Techniques and Functional Results

**DOI:** 10.7759/cureus.83465

**Published:** 2025-05-04

**Authors:** Manahil Awan, Sahib Memon, Kashif Memon, Jarallah H.J. Alkhazendar, Usama Shafique, Shahzad Ahmad

**Affiliations:** 1 General Practice, Liaquat National Hospital, Karachi, PAK; 2 Orthopedics and Traumatology, Stoke Mandeville Hospital, Aylesbury, GBR; 3 Trauma and Orthopedics, Queen Elizabeth Hospital Birmingham, Birmingham, GBR; 4 General and Emergency Surgery, East and North Hertfordshire NHS Trust - Lister Hospital, Stevenage, GBR; 5 General Surgery, Azra Naheed Medical College, Lahore, PAK; 6 Surgery, Liaquat National Hospital, Karachi, PAK

**Keywords:** biceps tenodesis, fixation method, functional outcome, shoulder, subpectoral, suprapectoral, systematic review

## Abstract

This systematic review compares the clinical outcomes of suprapectoral versus subpectoral biceps tenodesis performed using various fixation techniques for the treatment of long head of the biceps tendon pathology. A structured search identified eligible randomized controlled trials and meta-analyses that reported functional outcomes such as the American Shoulder and Elbow Surgeons Score (ASES), Constant, and Single Assessment Numeric Evaluation (SANE) scores. The included studies demonstrated no significant differences in postoperative functional outcomes between the two surgical approaches. However, there was a consistent trend indicating a higher complication rate associated with the suprapectoral technique. These findings suggest that while both approaches are effective in restoring function, subpectoral tenodesis may be a safer and more reliable option in appropriate patient populations.

## Introduction and background

Biceps tenodesis is a widely performed surgical intervention for managing pathology of the long head of the biceps tendon, particularly in patients with superior labrum anterior and posterior (SLAP) lesions, tendinopathy, or partial tearing [1]. As surgical techniques have evolved, two primary anatomical approaches have gained prominence: suprapectoral and subpectoral biceps tenodesis. The suprapectoral approach typically involves arthroscopic techniques and is performed proximal to the pectoralis major tendon insertion, whereas the subpectoral approach is often carried out through a mini-open incision distal to the pectoralis major. Each technique offers unique advantages - suprapectoral tenodesis allows for a minimally invasive route and may minimize soft tissue dissection, while subpectoral tenodesis is believed to reduce the risk of persistent groove pain and may offer a more anatomic tensioning of the tendon [2].

Alongside the choice of surgical approach, fixation technique plays a critical role in clinical outcomes. Surgeons may use a variety of devices, such as interference screws, suture anchors, or cortical buttons, each offering different biomechanical properties and healing environments [3]. However, despite the increasing utilization of these methods, the optimal combination of approach and fixation technique remains unclear, with current literature presenting varied and sometimes conflicting results. Questions persist regarding complication rates, reoperation frequency, residual pain, and functional recovery between suprapectoral and subpectoral approaches, especially when performed with different fixation strategies.

Given the growing volume of surgical interventions and the clinical significance of ensuring optimal patient outcomes, it is essential to systematically compare these approaches. The present review focuses on synthesizing the highest quality available evidence - randomized controlled trials and meta-analyses - to evaluate and compare clinical outcomes, complications, and revision rates associated with suprapectoral versus subpectoral biceps tenodesis, particularly in the context of varying fixation techniques.

This systematic review was guided by a structured PICO framework [4] to ensure clinical relevance and methodological clarity. The Population (P) includes adult patients undergoing surgical treatment for long head of the biceps tendon pathology. The Intervention (I) comprises suprapectoral biceps tenodesis, performed with various fixation techniques such as interference screws, anchors, or buttons. The Comparison (C) is subpectoral biceps tenodesis, also utilizing a variety of fixation modalities. The Outcomes (O) of interest include clinical outcomes (pain relief, function), postoperative complications (e.g., infection, tendon rupture), reoperation or revision rates, and patient-reported outcome measures. By framing the research question through the PICO structure, this review aims to generate clear and applicable evidence to inform surgical decision-making and improve patient care.

## Review

Materials and methods

Search Strategy

A comprehensive literature search was conducted in accordance with PRISMA (Preferred Reporting Items for Systematic Reviews and Meta-Analyses) guidelines [5] to ensure methodological rigor and transparency. We systematically searched four major electronic databases: PubMed (MEDLINE), Embase, Scopus, and the Cochrane Library, from database inception to August 2024. The search strategy incorporated combinations of keywords and MeSH terms including "biceps tenodesis," "suprapectoral," "subpectoral," "interference screw," "fixation technique," and "clinical outcomes." Filters were applied to include only randomized controlled trials and meta-analyses published in English. After removal of duplicates and screening of titles, abstracts, and full texts, three high-quality studies were selected for final inclusion: two randomized controlled trials and one systematic review with meta-analysis, each comparing suprapectoral and subpectoral biceps tenodesis using various fixation methods.

Eligibility Criteria

Studies were eligible for inclusion if they directly compared suprapectoral and subpectoral biceps tenodesis techniques in the management of long head of the biceps tendon pathology. Only randomized controlled trials (RCTs) and systematic reviews with meta-analyses published in English were considered to ensure high-quality evidence. Studies had to report on at least one of the following outcomes: functional scores (ASES, Constant, SANE), strength, range of motion, or revision rates. Exclusion criteria included non-comparative studies, case reports, cadaveric or biomechanical studies, and articles without full-text access or lacking quantitative outcome data relevant to clinical endpoints.

Data Extraction

Data were independently extracted from each included study using a standardized data extraction table. Key variables collected included study design, sample size, surgical approach (suprapectoral vs. subpectoral), fixation technique used, duration of follow-up, outcome measures, and key findings. For randomized controlled trials, the fixation method was confirmed to be consistent (PEEK interference screws), allowing for an isolated comparison of surgical approach. In the case of the meta-analysis, pooled results from both RCTs and non-RCTs were considered, with special attention paid to statistically significant outcomes, including complication rates and functional scores.

Data Analysis and Synthesis

Given the limited number of included studies and heterogeneity in study design and outcome reporting, a qualitative synthesis was performed. Results from the two RCTs were reviewed narratively, highlighting similarities in functional outcomes and differences in operative time and complication profiles. The 2025 meta-analysis was used to supplement findings with quantitative data, including odds ratios and confidence intervals, particularly for complication rates. The synthesis focused on aligning statistical significance with clinical relevance, emphasizing areas of consensus while acknowledging limitations such as sample size variability and methodological differences among the included studies.

Results

Study Selection Process

**Figure 1:**
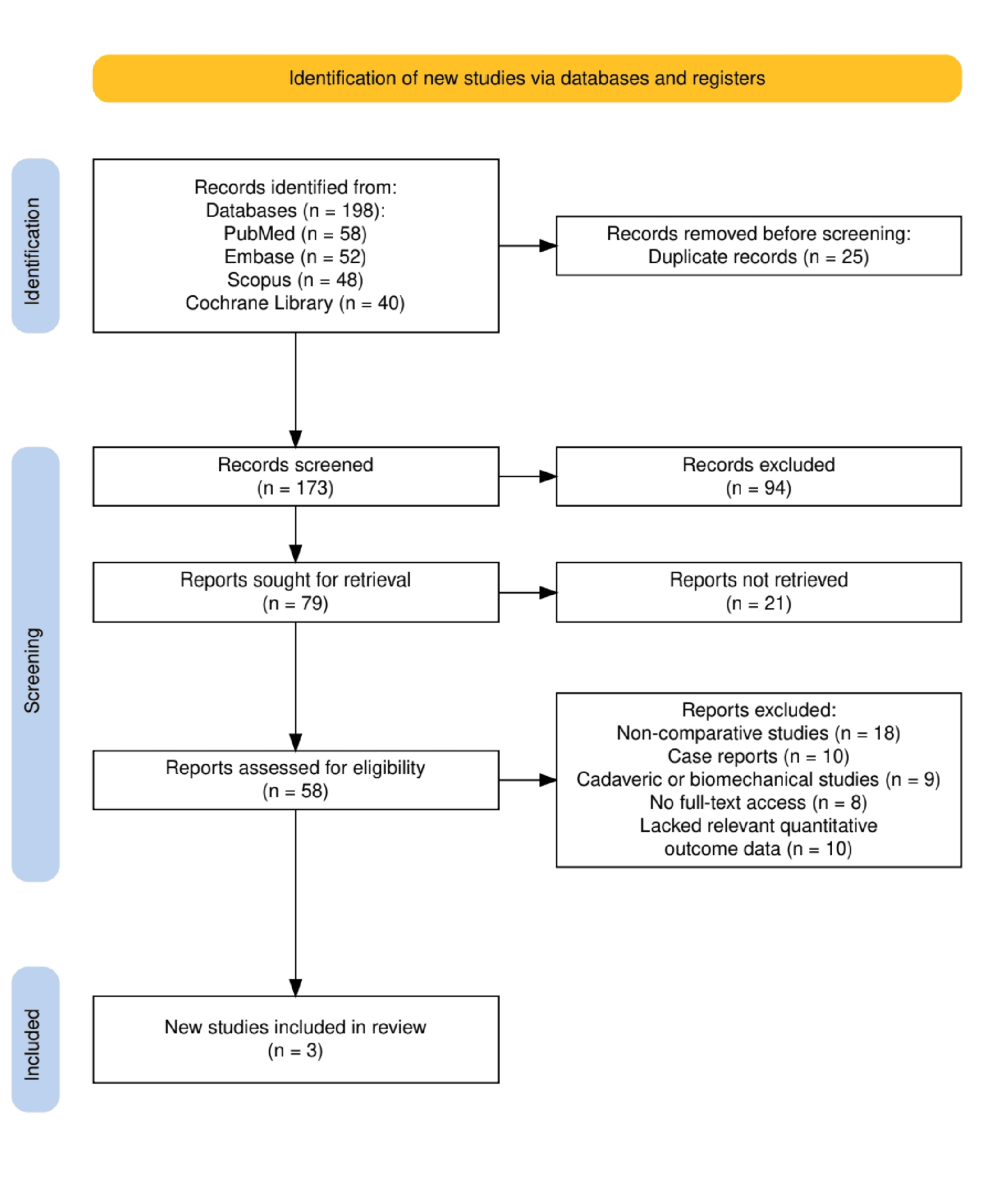
PRISMA flowchart represents the study selection process.

As illustrated in Figure 1, a total of 198 records were identified through systematic searches of four databases: PubMed (58), Embase (52), Scopus (48), and the Cochrane Library (40). After the removal of 25 duplicate records, 173 studies remained for screening. Following title and abstract screening, 94 records were excluded, and 79 full-text articles were sought for retrieval. Of these, 21 could not be retrieved, leaving 58 articles for full-text eligibility assessment. Ultimately, 55 studies were excluded due to being non-comparative (18), case reports (10), cadaveric or biomechanical studies (9), lack of full-text access (8), or absence of relevant quantitative outcome data (10). Three high-quality studies - two randomized controlled trials and one systematic review with meta-analysis - met the inclusion criteria and were included in the final review.

Characteristics of the Selected Studies

As summarized in Table 1, the included studies consisted of two randomized controlled trials and one meta-analysis, all comparing suprapectoral and subpectoral biceps tenodesis. Both randomized trials utilized PEEK interference screws and compared arthroscopic suprapectoral with open subpectoral techniques. One study involved 75 patients with follow-up assessments at three, six, and 12 months, showing no significant differences in ASES, Constant, or SANE scores, though the suprapectoral approach required longer operative time. The second trial followed 73 patients for a minimum of two years and again found no statistically significant differences in outcomes or revision rates, reinforcing the equivalence in functional recovery between approaches. The meta-analysis, which incorporated 13 studies with varying fixation methods and follow-up durations, reported comparable functional outcomes but identified a significantly higher complication rate associated with the suprapectoral approach (OR 2.65, p = 0.0002), leading to a recommendation in favor of subpectoral tenodesis when feasible.

**Table 1:** Characteristics of the Selected Studies ASES – American Shoulder and Elbow Surgeons Score; SANE – Single Assessment Numeric Evaluation; ROM – Range of Motion; UCLA – University of California at Los Angeles Shoulder Score; SST – Simple Shoulder Test; VAS – Visual Analog Scale (commonly used for pain measurement); PEEK – Polyether Ether Ketone (a biocompatible plastic used for implants); ASPBT – Arthroscopic Suprapectoral Biceps Tenodesis; OSPBT – Open Subpectoral Biceps Tenodesis; RCT – Randomized Controlled Trial

Study (Author, Year)	Study Design	Sample Size	Surgical Approach (Supra/Sub)	Fixation Method	Follow-up Duration	Outcome Measures	Key Findings
Forsythe et al., 2020 [6]	RCT	n = 75 (ASPBT = 37, OSPBT = 38)	Arthroscopic Suprapectoral vs Open Subpectoral	PEEK interference screw	3, 6, and 12 months	ASES, Constant score, SANE, ROM, strength	No significant differences in outcomes or strength; ASPBT had longer surgical time; both groups showed clinically meaningful ASES improvement
Gahlot et al., 2025 [7]	Meta-analysis	13 studies (3 RCTs, 10 non-RCTs)	Suprapectoral vs Subpectoral	Mixed fixation methods	Varies across studies	ASES, Constant score, UCLA, SST, VAS, ROM	Comparable functional outcomes; suprapectoral associated with significantly higher complication rate (OR 2.65, p = 0.0002); subpectoral preferred
Forsythe et al., 2022 [8]	RCT	n = 73 (ASPBT = 37, OSPBT = 36)	Arthroscopic Suprapectoral vs Open Subpectoral	PEEK interference screw	Minimum 24 months (mean 2.9 years)	ASES, Constant score, SANE	No significant differences in outcomes at any time point; both groups maintained functional improvement; no revisions required

Quality Assessment

As presented in Table 2, the quality of the included studies was evaluated using appropriate tools based on study design. Both randomized controlled trials by Forsythe et al. [6,8] were assessed using the Cochrane Risk of Bias 2 (RoB 2) tool [9] and were rated as having low risk of bias across all domains, including randomization, deviations from intended interventions, missing outcome data, outcome measurement, and selective reporting. This reflects their strong methodological integrity and consistent reporting standards. The meta-analysis by Gahlot et al. [7] was assessed using the AMSTAR 2 tool [10] and received a moderate quality rating, primarily due to its inclusion of both RCTs and non-RCTs and limited reporting on heterogeneity assessments. Nonetheless, it maintained low concerns for reporting bias and missing data, supporting its overall reliability as a synthesis of current evidence.

**Table 2:** The quality of the included studies RCT – Randomized Controlled Trial; Non-RCTs – Non-Randomized Controlled Trials; ASPBT – Arthroscopic Suprapectoral Biceps Tenodesis; OSPBT – Open Subpectoral Biceps Tenodesis; Suprapectoral – Above the pectoralis major muscle; Subpectoral – Below the pectoralis major muscle; ASES – American Shoulder and Elbow Surgeons Score; SANE – Single Assessment Numeric Evaluation; ROM – Range of Motion; UCLA – University of California at Los Angeles Shoulder Score; SST – Simple Shoulder Test; VAS – Visual Analog Scale; OR – Odds Ratio

Study (Author, Year)	Study Design	Quality Assessment Tool	Randomization Process	Deviations from Intended Interventions	Missing Outcome Data	Outcome Measurement	Selective Reporting	Overall Quality Rating
Forsythe et al., 2020 [6]	RCT	RoB 2	Low risk	Low risk	Low risk	Low risk	Low risk	Low Risk of Bias
Gahlot et al., 2025 [7]	Meta-analysis	AMSTAR 2	Moderate (mixed RCT/non-RCT)	Not applicable	Low concern	Moderate (limited reporting of heterogeneity tests)	Low concern	Moderate Quality
Forsythe et al., 2022 [8]	RCT	RoB 2	Low risk	Low risk	Low risk	Low risk	Low risk	Low Risk of Bias

Discussion

Our systematic review, which included two randomized controlled trials and one meta-analysis, found no statistically significant differences in functional outcomes between arthroscopic suprapectoral and open subpectoral biceps tenodesis. In the 2020 and 2022 RCTs by Forsythe et al. [6,8], both groups showed comparable improvements in ASES, Constant, and SANE scores at 12-month and midterm follow-ups (P-values > 0.05 across all outcomes), with no revision surgeries required. However, the 2025 meta-analysis by Gahlot et al. [7], which synthesized 13 studies, reported a significantly higher complication rate associated with suprapectoral tenodesis (OR 2.65; 95% CI 1.57-4.45; P = 0.0002), despite similar outcome scores such as ASES, UCLA, and VAS. These findings suggest that while both approaches are functionally effective, subpectoral tenodesis may offer a safer profile, particularly in reducing postoperative complications.

Previous literature has produced mixed results regarding the optimal location for biceps tenodesis. Some earlier studies have reported no clinically significant differences between suprapectoral and subpectoral approaches [11], aligning closely with the findings of Forsythe et al. [6,8] included in our review. However, other reports have suggested a trend toward fewer mechanical complications, such as tendon rupture or groove pain, in subpectoral tenodesis. The 2025 meta-analysis by Gahlot et al. [7] adds to this perspective by statistically reinforcing the association between suprapectoral techniques and a higher complication rate. This consistency with prior reports strengthens the argument that subpectoral tenodesis, while functionally similar, may carry a lower risk of adverse outcomes, particularly when technical execution or fixation method varies.

The observed differences in complication rates may be attributed to several biomechanical and technical factors. Suprapectoral tenodesis is performed more proximally, often within or near the bicipital groove, where persistent friction and poor tendon-bone healing may predispose to anterior shoulder pain or tendon failure [12]. Additionally, arthroscopic suprapectoral procedures demand higher technical precision and may be more susceptible to tendon tensioning errors or inadequate fixation due to the curved anatomy of the groove [13]. In contrast, subpectoral tenodesis offers a more predictable exposure and allows fixation in a well-vascularized metaphyseal region, potentially enhancing tendon incorporation. The use of the same fixation method (PEEK interference screws) in both groups across Forsythe’s trials neutralized device-based variability, highlighting that surgical approach alone may influence complication rates. Furthermore, surgeon experience and learning curve may play a subtle role, particularly in arthroscopic suprapectoral procedures which require advanced visualization skills.

These findings have important implications for clinical decision-making. While both suprapectoral and subpectoral approaches yield comparable improvements in functional outcomes, the significantly higher complication rate associated with suprapectoral tenodesis suggests that subpectoral fixation may be the more reliable and safer option [14], particularly in patients at higher risk for mechanical failure or reoperation. For surgeons selecting a technique, factors such as tendon quality, anatomical variation, and operative visibility must be considered. In high-demand or athletic patients, where durability and low recurrence are critical, the subpectoral approach may offer more consistent long-term benefits [15]. These insights can guide individualized surgical planning and contribute to optimizing outcomes in shoulder surgery.

A key strength of this review lies in its adherence to rigorous methodological standards, including PRISMA guidelines for study selection, screening, and reporting. By limiting inclusion to randomized controlled trials and high-quality meta-analyses, we ensured that the findings are grounded in strong levels of evidence. The consistent use of standardized outcome measures such as ASES, Constant, and SANE scores across studies enhanced comparability, while fixation method uniformity in two of the RCTs helped isolate the influence of surgical approach alone. The inclusion of both short-term and midterm follow-up periods further adds clinical value, offering insight into the durability of outcomes over time.

However, certain limitations must be acknowledged. First, the overall number of high-quality RCTs available on this topic remains limited, and the included studies had modest sample sizes, potentially underpowering subgroup analyses. The 2025 meta-analysis included heterogeneous studies, many of which were retrospective or varied in fixation techniques, which may have introduced bias despite pooled analysis. Additionally, long-term outcomes beyond three years remain largely unexplored, and patient-centered outcomes such as satisfaction, cosmetic concerns, and time to return to function were not consistently reported. Future research should focus on large-scale, multicenter RCTs comparing specific fixation methods across both approaches, with extended follow-up and stratification by activity level, age, and tendon pathology. Incorporating cost-effectiveness analyses and patient-reported quality of life metrics will also be crucial for refining surgical decision-making.

## Conclusions

In this systematic review, both suprapectoral and subpectoral biceps tenodesis techniques demonstrated comparable functional outcomes; however, evidence indicates that suprapectoral tenodesis is associated with a higher complication rate. Based on current high-quality data, subpectoral tenodesis may be the preferred approach when prioritizing surgical safety and reliability without compromising clinical efficacy.
